# Intratumoral PD-1^+^CD8^+^ T cells associate poor clinical outcomes and adjuvant chemotherapeutic benefit in gastric cancer

**DOI:** 10.1038/s41416-022-01939-8

**Published:** 2022-08-24

**Authors:** Kuan Yu, Yun Gu, Puran Zhang, Hanji Fang, Yifan Cao, Jieti Wang, Chao Lin, Hao Liu, Heng Zhang, Hongyong He, Ruochen Li, Jing Qin, He Li, Jiejie Xu

**Affiliations:** 1 Department of General Surgery, Zhongshan Hospital, Fudan University, Shanghai, China; 2 NHC Key Laboratory of Glycoconjugate Research, Department of Biochemistry and Molecular Biology, School of Basic Medical Sciences, Fudan University, Shanghai, China; 3 Department of Gastric Surgery, Fudan University Shanghai Cancer Center, Shanghai, China

**Keywords:** Gastric cancer, Cancer microenvironment, Tumour immunology

## Abstract

**Background:**

Although PD-1 has been reported to be a marker of T-cell exhaustion in several malignancies, the biological role of PD-1^+^CD8^+^ T cells in gastric cancer (GC) remains unclear. Herein, we aimed to investigate the role of PD-1^+^CD8^+^ T cells in the tumour microenvironment and its clinical significance in GC.

**Designs:**

This study included 441 tumour microarray specimens and 60 Flow cytometry specimens of GC patients from Zhongshan Hospital, and 250 GC patients from *the Asian Cancer Research Group*.

**Results:**

Here, we demonstrated that PD-1^+^CD8^+^ T cells functioned as an independent adverse prognosticator in GC. In addition, an abundance of intratumoral PD-1^+^CD8^+^ T cells indicated worse chemotherapeutic responsiveness to fluorouracil in Stage III GC patients. Mechanistically, PD-1^+^CD8^+^ T cell high infiltration indicated an exhausted phenotype of global CD8^+^ T cells in GC tissues, which was characterised by elevated immune checkpoint expression including CTLA-4 and TIM-3, whereas decreased expression of perforin. Furthermore, PD-1^+^CD8^+^ T cell high-infiltration patients with Stage III GC held elevated activity of several therapeutic signal pathways.

**Conclusions:**

Our study highlighted that PD-1^+^CD8^+^ T cell abundance predicts inferior prognosis in GC, and may serve as a novel predictive biomarker to guide therapeutic option.

## Introduction

Gastric cancer (GC) ranks the fifth most frequently diagnosed malignancies and the third major cause of cancer-related mortality worldwide [1–4]. Generally, radical gastrectomy remains the only curative treatment for GC [5, 6]. Nevertheless, since early-stage GC is asymptomatic, most patients are diagnosed at an advanced stage with dismal clinical outcomes [4]. Adjuvant chemotherapy (ACT) has been routinely applied in advanced-stage GC patients to overcome postoperative recurrence [4], of which 5-fluorouracil (5-FU) has been recognised as one of the standard-of-care agents [7–9]. However, restricted survival benefit was achieved due to intrinsic and acquired chemoresistance [10, 11]. Hence, novel GC stratification frameworks to facilitate personalised treatment selection have entered the spotlight.

Tumours are organised ecosystems instead of the simply sum of subclones. Tumour microenvironment (TME) is the “fertile soil” for cancer initiation and progression, which plays a versatile role in determining the biological properties of cancer [12]. Specifically, the immune contexture represents a crucial component of TME, which is shaped by the density, composing, functional status and reciprocal interaction of the tumour-infiltrating immune cells [13]. As we have previously reported, the immune contexture in GC is predictive of both overall prognosis and response to either chemotherapy or immunotherapy [14]. However, while effector immune subsets are responsible for immune scrutiny, tumour cells may also manipulate the host immune system, cause immune evasion and lead to disease progression [15]. As a result, the abundance of several immune subsets, including special subsets of macrophages [16, 17], CD8^+^ T cells [18] and of CD4^+^ T cells [19, 20], may even relate to a tumour-promoting TME.

Usually, CD8^+^ T cells are paramount in the antitumour immune process [21]. Nevertheless, existing studies that examine the prognostic impact of CD8^+^ T cell infiltration in GC are inconsistent [22], indicating the heterogeneity of intratumoral CD8^+^ T cells. Contrasting to conventional activated CD8^+^ T cells that function as the immune effector, persistent antigen stimulation may also lead to a dysfunctional state called T-cell exhaustion [23, 24]. Immune checkpoints are typically overexpressed on exhausted T cells, of which programmed cell death-1 (PD-1) has been recognised as a crucial marker with biological effects to inhibit T-cell activation. Recent studies have revealed that the abundance of PD-1^+^CD8^+^ T cells is associated with worse prognosis and impaired antitumour immunity in patients with liver [25], lung [26], ovary [27], colorectum [28] and bladder [29] malignancies. Interestingly, the latest research reported that PD-1 might not indicate CD8^+^ T cell dysfunction in GC [30]. However, the clinical impact of PD-1^+^CD8^+^ T cells in GC remains largely unknown. Thus, we sought to delve into the predictive significance of PD-1^+^CD8^+^ T cells through the comprehensive analysis of large-scaled retrospective cohorts.

In this study, we found that PD-1^+^CD8^+^ T cell infiltration could identify a subgroup of GC with poor prognosis and inferior responsiveness to ACT with immunosuppressive contexture and CD8^+^ T cell dysfunction.

## Methods

### Patients and tissue samples

Three independent GC cohorts were enrolled in this study. The Zhongshan Hospital (ZSHS) Cohort originally contained 496 GC patients from Zhongshan Hospital, Fudan University (Shanghai, China), whereas 55 patients were excluded for clinical data missing, metastatic diseases and dot loss. The remaining 441 patients were available for further analysis. In ZSHS Cohort, 441 patients were randomly divided into two independent sets, discovery set (*n* = 200) and validation set (*n* = 241). The clinicopathological characteristics of enrolled patients are listed in Table 1. The clinical tumour stages were determined referring to the 7th Edition of the American Joint Committee on Cancer (AJCC) Cancer Staging System. After gastrectomy, 5-fluorouracil-based ACT was given to TNM Stage II and III patients for at least one therapeutic cycle (median: six cycles; range: 1–8 cycles). Two endpoints were included in this study, including the overall survival (OS) and disease-free survival (DFS). OS and DFS referred to the period from gastrectomy to death, or time from surgery to first recurrence, respectively. Patients were observed until April 2014. Transcriptomic and clinical data of Asian Cancer Research Group (ACRG) Cohort were downloaded in January 2019 (https://www.ncbi.nlm.nih.gov/geo/query/acc.cgi?acc=GSE62254). Fifty out of the overall 300 GC patients were excluded due to data incompleteness. The remaining 250 patients were eligible for subsequent analysis. In addition, the Flow Cytometry (FCM) Cohort included 60 GC patients with fresh tumour specimens. Those patients underwent gastrectomy in Zhongshan Hospital, Fudan University (Shanghai, China) during September 2018 to July 2019. This study has been approved by the Clinical Research Ethics Committee of Zhongshan Hospital, Fudan University. Written informed consents were obtained from patients of ZSHS Cohort or FCM Cohort.

**Table 1:** Relationship between PD-1^+^CD8^+^ T cell infiltration and clinical characteristics.

Factors	Discovery data set			Validation data set		
	PD-1^+^CD8^+^ T cells infiltration			PD-1^+^CD8^+^ T cells infiltration		
	Low	High	*P* value	Low	High	*P* value
All patients	105	95		118	123	
Age (years)^a^			0.130			0.227
Median (IQR)	61 (52–70)	59 (53–67)		60 (51–69)	59 (53–70)	
Gender			0.521			0.927
Female	32	33		32	34	
Male	73	62		86	89	
Localisation			0.825			0.136
Proximal	27	21		31	30	
Middle	16	16		10	21	
Distal	62	58		77	72	
Tumour size (cm)^a^			0.908			0.707
Median (IQR)	3.0 (2.0–5.0)	4.0 (3.0–5.0)		3.5 (2.0–4.5)	3.5 (2.0–5.0)	
Grade			0.569			0.205
G1	4	4		8	7	
G2	24	16		18	30	
G3&G4	77	75		92	86	
Lauren classification			0.762			0.963
Diffuse	42	40		39	41	
Intestinal	63	55		79	82	
T classification			**0.050**			0.368
T1	22	10		29	25	
T2	18	10		15	20	
T3	20	18		25	18	
T4	45	57		49	60	
N classification			0.317			0.613
N0	44	34		51	43	
N1	12	8		13	17	
N2	23	18		20	23	
N3	26	35		34	40	
TNM stage			0.100			0.487
I	28	14		39	32	
II	28	26		22	25	
III	49	55		57	66	
Adjuvant chemotherapy^b^			0.100			**0.035**
No	45	30		62	48	
Yes	60	65		56	75	

*PD-1* programmed cell death protein 1, *TNM* tumour node metastasis.

^a^Modeled as a continuous variable.

^b^Patients with adjuvant chemotherapy received at least one cycle of 5-fluoruracil-based chemotherapy.

*P* value < 0.05 marked in bold font shows statistical significance.

### Immunohistochemistry (IHC)

Formalin-fixed, paraffin-embedded tissue microarray (TMA) as described previously. The double-stained IHC was performed as follows. The IHC antibodies are listed in Supplementary Table 1. In brief, the slides were baked at 60 °C for 6 h, deparaffinized in xylene three times (15 min each) and rehydrated in graded alcohol. Next, the slides were immersed in Antigen Retrieval Buffer (100× Tris-EDTA Buffer, pH 9.0) for antigen retrieval, blocked with 3% H_2_O_2_ in methanol at 37 °C for 30 min and then incubated with 10% normal goat serum at 37 °C to eliminate nonspecific reactions. For single IHC staining, the slides were incubated with the primary antibodies at 4 °C overnight and PD-1 was visualised by 3,3′-diaminobenzidine (DAB) stain system. For double IHC staining, after being processed as the same of single IHC DAB staining, the slides were incubated with the second primary antibodies at 4 °C for 2 h, and CD8 was revealed in red colour using the Bond Polymer Refine Red Detection Kit (Leica Biosystems).

### Evaluation of IHC

All TMA slides were scored independently under Leica DM6000 B Microsystems by two pathologists (Dr. Lingli Chen and Dr. Yunyi Kong), who were blinded to clinicopathological data. The positive cells were enumerated from the representative view of the three sections in the high-power field (HPF, ×200 magnification). The mean value of their evaluations is defined as the formal cell density. The cut-off value was automatically determined by X-tile V.3.6.1 (Yale University). For PD-1^+^CD8^+^ T cells density, ≤7/HPF (at ×200 magnification) was defined as low, and ≥8/HPF was defined as high.

### FCM

Fresh tumour tissues were collected during the surgery. Single-cell suspension was performed as described [31]. Then tissues were stained with the indicated mAbs (30 min at 4 °C) after lysing red blood cells. According to the instructions given by the manufacturer, cells were stained with interested surface markers. Especially, the Fixation/Permeabilization Solution Kit (BD Biosciences) was used for intracellular molecules. Stained cells were washed and resuspended in phosphate-buffered saline/0.1% bovine serum albumin coupled with azide. Flow cytometry was performed using an BD celesta and analysed by FlowJo software (Tree Star, San Carlos, CA, USA).

### Bioinformatics

In brief, we first obtained a marker gene set of PD-1^+^CD8^+^ T cells, which was composed of PDCD1, CD8A, GZMA, SEMA4C, TGFBR1, FYN, ATP2B4, FAM129A, LPP, GAB3, CHST12, THAP8, AHNAK2, MEX3B, CD200, SALL2 and PALMDA [25]. Then, we used the gene set variation analysis (GSVA) R package to constitute the GSVA scores of the marker gene set for each sample in ACRG set. The GSVA score represented the degree of absolute enrichment of the PD-1^+^CD8^+^ T cells marker gene set in each sample.

### Statistical analysis

Descriptive statistics were used to summarise patients’ baseline characteristics and disease factors, including PD-1^+^CD8^+^ T cells infiltration. Results are shown as mean ± SD, and Student’s *t* test, Mann–Whiney *U* test, Wilcoxon signed-rank test, *χ*^2^ test and Spearman correlation analysis were used in this study. Kaplan–Meier curves were constructed to determine OS and DFS of patient subgroups and were evaluated by log-rank tests. Multivariate analyses of the Cox regression model were applied to estimate HRs and 95% CIs. Covariate effects on survival and interactions between different covariates were detected by the Cox proportional hazards regression model. All statistical analyses were performed using IBM SPSS Statistics v25.0 (Macintosh Version, IBM Corporation, NY, USA), MedCalc 15.6.1 (MedCalc Software bvba, Ostend, Belgium), GraphPad Prism v8.4.0 (Macintosh Version, GraphPad Software, California, USA), and R V.3.5.1. A two-tailed *P* value of *P* < 0.05 was considered statistically significant in our study.

## Results

### Intratumoral PD-1^+^CD8^+^ T cells were accumulated in GC and correlated with tumour progression

**Fig. 1:**
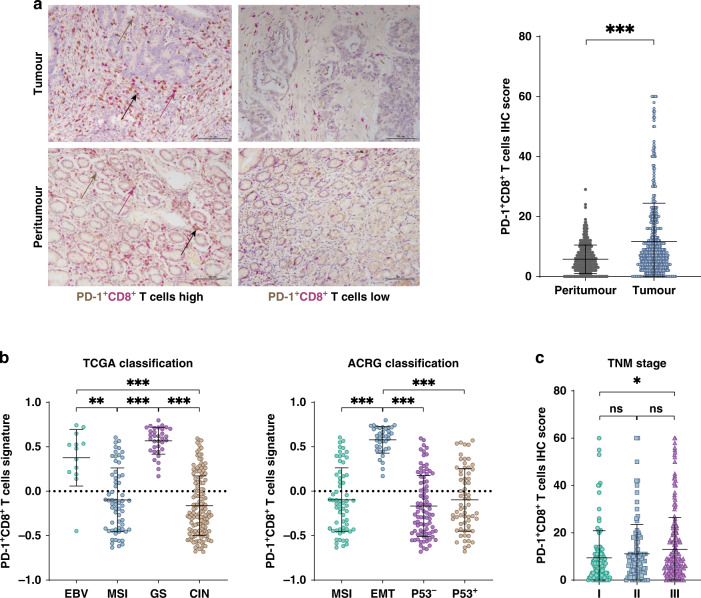
Intratumoral PD-1^+^CD8^+^ T cells were accumulated in gastric cancer and correlated with tumour progression. **a** Left: representative immunohistochemistry images for tumour-infiltrating PD-1^+^CD8^+^ T cells in gastric tissues. PD-1^+^ cells were stained in brown, while CD8^+^ cells were stained in red. Cells double-stained in brown and red were recognised as PD-1^+^CD8^+^ T cells (black arrows). Magnification: ×200; Scale bar, 100 μm. Right: scatterplots indicated the cumulative frequency of PD-1^+^CD8^+^ T cells in the gastric tumour and peritumor tissues (*n* = 441, Paired *t* test, *P* < 0.05). **b** PD-1^+^CD8^+^ T signature score in the EBV, MSI, GS and CIN subgroups (TCGA classification) and in the MSI, MSS/EMT, MSS/TP53^−^ and MSS/TP53^+^ subgroups (ACRG classification). **c** Association between PD-1^+^CD8^+^ T cells and tumour TNM stage was examined based on IHC staining (*P* = 0.049, One-way ANOVA followed by Tukey multiple comparisons). CIN Chromosomal instability, EBV EBV-positive, GS genomically stable, MSI microsatellite instable, MSS/EMT microsatellite stable and epithelial-to-mesenchymal transition, MSS/TP53^+^ microsatellite stable and tumour protein 53 active, MSS/TP53^−^ microsatellite stable and tumour protein 53 inactive. Small horizontal lines indicate the mean (±SD). *P < 0.05, **P < 0.01, ***P < 0.001, ns refers to not significant. ANOVA analysis of variance, PD-1 programmed cell death protein 1, SD standard deviation.

To examine the expression of PD-1 on CD8^+^ T cells in GC patients, we first performed double IHC staining in TMAs of ZSHS Cohort (Fig. 1a). As a result, cells double-stained in brown and red were recognised as PD-1^+^CD8^+^ T cells. Compared with peritumor tissues, PD-1^+^CD8^+^ T cells were more abundant in intratumoral tissues (*P* < 0.001; Fig. 1a). Therefore, we sought to provide biological insights and clinical relevance of PD-1^+^CD8^+^ T cells infiltrated in the TME. TCGA and ACRG subtyping represent two of the mainstream molecular classification frameworks of GC [32, 33]. We found that PD-1^+^CD8^+^ T cells were specifically enriched in genomic stable (GS) subtype and EBV-positive subtype according to TCGA classification and epithelial-to-mesenchymal transition (EMT) subtype based on ACRG classification. (Fig. 1b and Supplementary Fig 2A, B). Furthermore, we found that compared with Stage I tumours, Stage III tumours were infiltrated with more PD-1^+^CD8^+^ T cells (Fig. 1c; *P* = 0.042). Together, these findings indicated that PD-1^+^CD8^+^ T cells abundance might identify a biologically aggressive subtype of GC.

### Intratumoral PD-1^+^CD8^+^ T-cell infiltration indicated unfavourable prognosis in GC

**Fig. 2:**
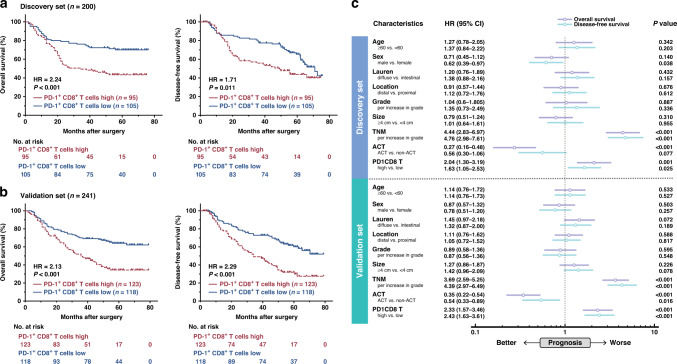
Intratumoral PD-1^+^CD8^+^ T cells infiltration yielded a poor prognosis in GC. **a**, **b** Kaplan–Meier curve of OS (left panel) and DFS (right panel) in discovery set (**a**) and validation set (**b**) according to PD-1^+^CD8^+^ T cells infiltration. Data were analysed by log-rank test. **c** Multivariate Cox analysis of OS and DFS for PD-1^+^CD8^+^ T cells infiltration and clinicopathologic factors in discovery set and validation set. HR hazard ratio, CI confidence interval, OS overall survival, DFS disease-free survival.

Consistent with previous studies, we found that the total infiltrating density of intratumoral CD8^+^ T cells in GC might not be indicative of prognosis (*P* = 0.135, Supplementary Fig. 1A). This conclusion suggests that relative to overall CD8^+^ T cell infiltration, specific functional subsets of CD8^+^ T cells may play a key role in GC. Here, we focused on intratumoral PD-1^+^CD8^+^ T cell in GC. Kaplan–Meier curves and log-rank test were conducted to compare the OS and DFS between PD-1^+^CD8^+^ T cells high and low infiltration subgroups. In both Discovery Set and Validation Set, abundance of PD-1^+^CD8^+^ T cells predicted significantly worse OS (*P* < 0.001 and *P* < 0.001; Fig. 2a, b, left panel) and DFS (*P* = 0.011 and *P* < 0.001; Fig. 2a, b, right panel). Subsequently, multivariate Cox regression analysis was performed. Clinicopathological parameters, including age, gender, Lauren’s classification, location, tumour grade, tumour size, and TNM stage, along with ACT and PD-1^+^CD8^+^ T cells density were incorporated into the multivariate Cox regression model. Consequently, we found that PD-1^+^CD8^+^ T cell infiltration predicted poor prognosis independent of the above clinicopathological parameters based on OS (discovery set: hazard ratio (HR) = 2.04, 95% confidence interval (CI) = 1.30–3.19, *P* = 0.001; validation set: HR = 1.63, 95% CI = 1.05–2.53, *P* = 0.025; Fig. 2c) and DFS (discovery set: HR = 2.33, 95% CI = 1.57–3.46, *P* < 0.001; validation set: HR: 2.43, 95% CI: 1.63–3.61, *P* < 0.001; Fig. 2c). Collectively, these results indicated that the infiltration of PD-1^+^CD8^+^ T cells could serve as an independent adverse prognosticator for survival outcomes in GC.

### Abundance of intratumoral PD-1^+^CD8^+^ T cells predicted inferior efficacy of ACT in TNM Stage III GC

**Fig. 3:**
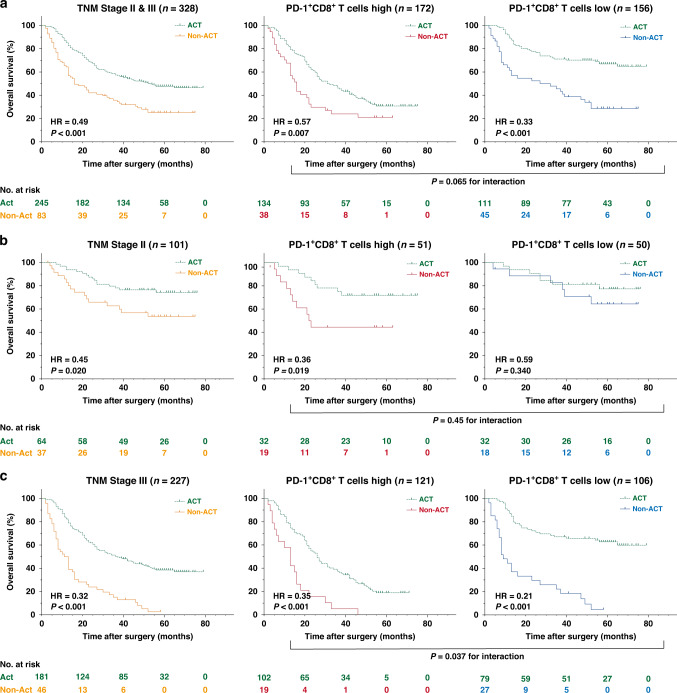
Relationship between PD-1^+^CD8^+^ T cell infiltration and responsiveness of 5-fluorouracil-based adjuvant chemotherapy. **a** Kaplan–Meier curves of OS in patients with TNM Stage II and III GC (n = 382, *P* < 0.001, HR = 0.49) (left panel), patients with high PD-1^+^CD8^+^ T cell infiltration (*n* = 172, *P* = 0.007, HR = 0.57) (middle panel) and patients with low PD-1^+^CD8^+^ T cells (*n* = 156, *P* < 0.001, HR = 0.33) (right panel) according to ACT application. Log-rank test was applied to Kaplan–Meier curves. **b** Kaplan–Meier curves of OS in patients with TNM Stage II GC (*n* = 101, *P* = 0.020, HR = 0.45) (left panel), patients with high PD-1^+^CD8^+^ T cell infiltration (*n* = 51, *P* = 0.019, HR = 0.36) (middle panel) and patients with high PD-1^+^CD8^+^ T cells (*n* = 50, *P* = 0.34, HR = 0.59) (right panel) according to ACT application. Log-rank test was applied to Kaplan–Meier curves. **c** Kaplan–Meier curves of OS in patients with TNM Stage III tumours (*n* = 227, *P* < 0.001, HR = 0.32) (left panel), patients with high PD-1^+^CD8^+^ T cell infiltration (*n* = 121, *P* < 0.001, HR = 0.36) (middle panel) and patients with high PD-1^+^CD8^+^ T cells (*n* = 106, *P* < 0.001, HR = 0.21) (right panel) according to ACT application. Log-rank test was applied to Kaplan–Meier curves.

In the ZSHS Cohort, patients with TNM II and III GC could benefit from fluorouracil-based ACT (*P* < 0.001, HR = 0.49; Fig. 3a). However, the predictive value of intratumoral PD-1^+^CD8^+^ T cells regarding ACT remains unclear. Thus, we aimed to explore the efficacy of fluorouracil-based ACT in different subgroups based on PD-1^+^CD8^+^ T cells infiltration with univariate Cox regression model. In patients with Stage II/III GC, superior ACT reactivity was shown in both PD-1^+^CD8^+^ T cells high subgroup (*P* = 0.007, HR = 0.57; Fig. 3a) and PD-1^+^CD8^+^ T cells low subgroup (*P* < 0.001, HR = 0.33; Fig. 3a). Therefore, in order to investigate the relationship between PD-1^+^CD8^+^ T cell infiltration and benefit from ACT, we further conducted an interaction analysis among subgroups, in which, a significant interaction test shows that the treatment effect significantly varies across the levels of the subgroup. Although no significant results were observed according to interaction test between PD-1^+^CD8^+^ T cells infiltration and ACT, in the PD-1^+^ CD8^+^ T cells high-infiltration subgroup, we observed a trend of poor response to chemotherapy (*P* = 0.065 for interaction; Fig. 3a). Consequently, we further stratified patients according to TNM stage, patients were divided into Stage II group (*P* = 0.020, HR = 0.45; Fig. 3b) and Stage III group (*P* < 0.001, HR = 0.32; Fig. 3c). In patients with Stage II GC, we found that ACT successfully prolonged OS in patients with PD-1^+^CD8^+^ T cells high infiltration (*P* = 0.019, HR = 0.36; Fig. 3b), while no survival benefit was observed in patients with low PD-1^+^CD8^+^ T cells infiltration (*P* = 0.34, HR = 0.59; Fig. 3b). However, the interaction test suggested that there was no significant difference in ACT effectiveness between the two groups of patients with Stage II GC (*P* = 0.45 for interaction; Fig. 3b). Therefore, we assumed that the negative results of PD-1^+^CD8^+^ T cells low infiltration patients with Stage II GC might result from the relatively small cohort (*n* = 50). Subsequently, in patients with Stage III GC, ACT provided a significant survival benefit in both PD-1^+^CD8^+^ T cells high-infiltration subgroup (*P* < 0.001, HR = 0.35; Fig. 3c) and PD-1^+^CD8^+^ T cells low-infiltration subgroup (*P* < 0.001, HR = 0.21; Fig. 3c). Interestingly, the interaction test revealed that PD-1^+^CD8^+^ T cells high-infiltration patients had worse therapeutic responsiveness to ACT than PD-1^+^CD8^+^ T cells low-infiltration patients (*P* = 0.037 for interaction; Fig. 3c). Herein, these results suggested that PD-1^+^CD8^+^ T cells low abundance predicted optimal fluorouracil-based chemotherapeutic responsiveness for patients with TNM Stage III GC, and patients with PD-1^+^CD8^+^ T cells high abundance might have a higher risk of chemoresistance.

### Intratumoral PD-1^+^CD8^+^ T cells abundance indicated impaired CD8^+^ T-cell effector function

**Fig. 4:**
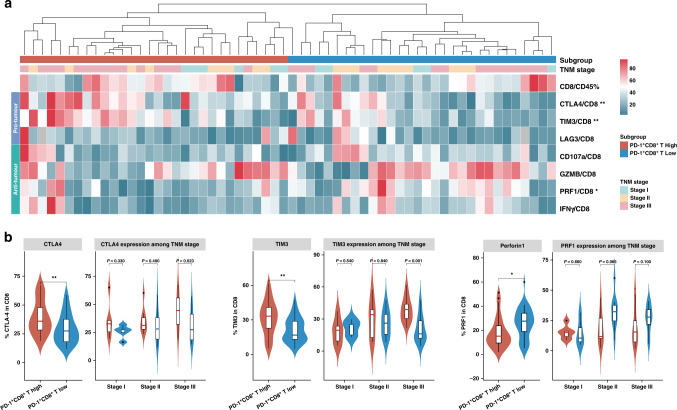
PD-1^+^CD8^+^ T cell abundance indicated T cell dysfunction. **a** Heatmap illustrating the infiltration of CD8^+^ T cells with Immune-related molecules high expression by flow cytometry analysis among PD-1^+^CD8^+^ T cell infiltration and clinical stage from patients with GC (normalised by the Z-score). **b** The quantification of exhausted markers (CTLA-4 and TIM-3) and effector cytokines (PRF1) expression on CD8^+^ T cells in PD-1^+^CD8^+^ T cells high/low abundance subgroups, comparisons were also demonstrated among different TNM stages. Data were analysed by Mann–Whitney *U* test. Small horizontal lines indicate the mean (± SD). *P < 0.05, **P < 0.01, ***P < 0.001, ns refers to not significant. All *P* values presented here were two-tailed. IFN-γ interferon-γ, GZMB granzyme B, PD-1 programmed cell death protein 1, CTLA-4 cytotoxic T-lymphocyte-associated protein-4, TIM-3 T cell immunoglobulin, LAG3 lymphocyte-activation gene 3.

Since we have highlighted the clinical significance of PD-1^+^CD8^+^ T cells, and found PD-1^+^CD8^+^ T cells could predict poor OS and inferior chemotherapeutic responsiveness in GC (Figs. 2 and  3), we wondered whether this subgroup of CD8^+^ T cells was associated with CD8^+^ T cell dysfunctional phenotype. Thus, FCM analysis was performed on sixty GC patients to detect the proportion of PD-1^+^CD8^+^ T cells among CD8^+^ T cells. Based on the above data, the sixty patients were divided into two subgroups (PD-1^+^CD8^+^ T cells high-infiltration and PD-1^+^CD8^+^ T cells low infiltration). The global characterisation of CD8^+^ T cells was subsequently investigated according to PD-1^+^CD8^+^ T cell abundance (Fig. 4a). Notably, we found that CD8^+^ T cells in tumours with PD-1^+^CD8^+^ T cells high infiltration expressed increased immune checkpoints, including cytotoxic T-lymphocyte-associated protein-4 (CTLA-4) and T-cell immunoglobulin domain and mucin domain-3 (TIM-3) while decreased effector cytokines, perforin 1 (PRF1) (Fig. 4b) than their counterparts. While no significant difference was observed among the two groups, regarding the proliferative ability (Ki-67), the effector cytokines, interferon-γ (INF-γ) and tumour necrosis factor-α (TNF-α) and cytotoxicity activation molecules, Granzyme B (GZMB) expressed by CD8^+^ T cells (Supplementary Fig. 3B). These findings indicated that terminally exhausted CD8^+^ T cells were most predominant in PD-1^+^CD8^+^ T cells high-infiltration Stage III GC, and high expression of PD-1 on CD8^+^ T cells were associated with counteracted and impaired CD8^+^ T cell antitumor immunity. The results aforementioned preliminarily verified our conjecture that intratumoral PD-1^+^CD8^+^ T cell abundance might contribute to immune suppression and dampen CD8^+^ T cell immune response in GC.

### Abundance of PD-1^+^CD8^+^ T cells correlated with actionable genomic alterations

Previous results we acquired had shown that Stage III GC patients with high PD-1^+^CD8^+^ T cell infiltration have a higher risk of chemoresistance. In order to improve the survival benefit of these patients, more appropriate treatment strategies need to be considered and may be used as an alternative after chemoresistance.

**Fig. 5:**
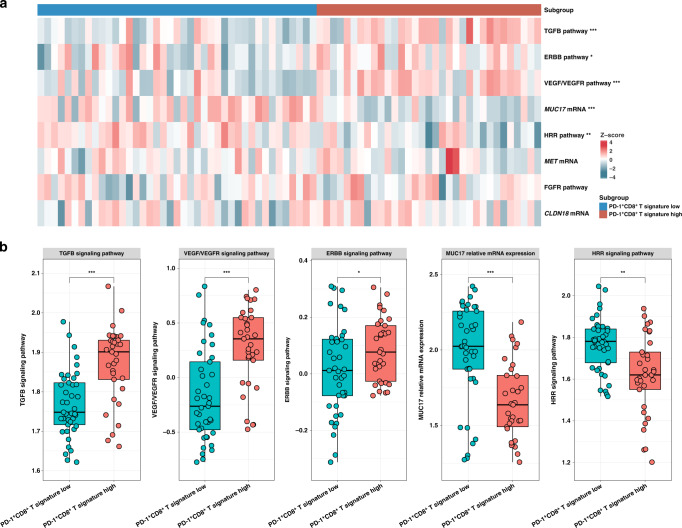
Association between PD-1^+^CD8^+^ T cell abundance and targetable genomic alterations in patients with Stage III gastric cancer. **a** Heatmap demonstrated the genomic alterations of potential therapeutic targets in GC based on PD-1^+^CD8^+^ T cell signature level. **b** Quantification analyses of therapy-associated signal pathway signature between PD-1^+^CD8^+^ T cell signature low/high-expression subgroup among Stage III GC patients in ACRG cohort: TGFB signalling pathway (*P* < 0.001), VEGF/VEGFR signalling network (*P* < 0.001), ERBB signalling pathway (*P* < 0.05), MUC17 relative mRNA expression (*P* < 0.001), HRR signalling pathway (*P* < 0.010); HRR, homologous recombination repair. Data were analysed by Mann–Whitney *U* test. Small horizontal lines indicate the mean (±SD). *P < 0.05, **P < 0.01, ***P < 0.001, ns refers to not significant. *P* values presented here were two-tailed.

We sought to inspect the association between PD-1^+^CD8^+^ T cell signature and potentially targetable genomic alterations in Stage III GC. Notably, PD-1^+^CD8^+^ T cells high subgroup exhibited significant gene enrichment of TGFB signalling, ERBB signalling (human epidermal growth factor receptor) signalling, VEGF/VEGFR (vascular endothelial growth factor) signalling (Fig. 5). The above findings may provide new therapeutic ideas for Stage III GC patients with high PD-1^+^CD8^+^ T cell infiltration who are resistant to chemotherapy. Meanwhile, We also found that PD-1^+^CD8^+^ T cells low subgroup exhibited significant gene enrichment MUC17 mRNA and HRR signalling. Taken together, our results suggest advanced patients with GC could be divided into two subgroups, which might be sensitive to different therapeutic strategies.

## Discussion

Previous results that examine the clinical significance of CD8^+^ T cells in GC are inconsistent, reflecting the functional complexity of this heterogenous cell population [34]. In this study, we delineated a functional distinct subset of PD-1 expressing CD8^+^ T cells in GC, and linked PD-1^+^CD8^+^ T abundance with immune-evasive TME and unfavourable clinical outcomes.

First, our study again highlighted the heterogeneity of CD8^+^ T cells in GC, and profiled the phenotypic and functional properties of PD-1 expressing CD8^+^ T subset. We found that PD-1^+^CD8^+^ T cells displayed a dysfunctional phenotype, featured by overexpression of immune checkpoints, including CTLA-4 and TIM-3, yet loss of perforin expression. Specifically, PD-1^+^CD8^+^ T cells were especially abundant in EMT/MSS subtype GC according to ACRG classification. The EMT/MSS subtype GC was reported to correlate with low TMB, worst prognosis and high risk of recurrence [32], which may partly account for why PD-1^+^CD8^+^ T cell high-infiltration tumours were extremely lethal. As a regulatory pathway to shape the EMT subtype, we also observed elevated TGF-beta signalling in PD-1^+^CD8^+^ T abundant tumours, which was found to correlate with worse clinical outcomes and immune-evasive TME in our previous work [14]. Consequently, dual inhibition of PD-1/PD-L1 axis and TGF-beta signalling may be especially rewarding in patients with PD-1^+^CD8^+^ T cells high infiltration.

Second, we provided PD-1^+^CD8^+^ T as a novel biomarker to select GC patients for both fluorouracil-based ACT and targeted agents. In this study, we found that TNM Stage III GC patients with PD-1^+^CD8^+^ T abundance could restrictedly benefit from fluorouracil-based ACT. Thus, PD-1^+^CD8^+^ T cells could be a predictive biomarker to select TNM Stage III GC patients for postoperative chemotherapy. Moreover, drugs targeting actionable genomic alterations [35], including HER2 inhibitors and anti-angiogenesis therapies, have been brought into clinical trials of advanced-stage GC [36, 37]. Our results revealed that tumours with PD-1^+^CD8^+^ T abundance displayed elevated VEGF/VEGFR and ERBB signalling pathway activity. Therefore, patients with this aggressive subtype GC might potentially benefit from Anti-angiogenesis and HER2 targeted therapies.

Although a previous study by Shen et al. showed that PD-1^+^CD8^+^ T cells showed equivalent function to their PD-1^-^CD8^+^ T cells counterparts and they did not predict tumour progression in GC, which seemed contradictory with our findings [30]. Actually, PD-1^+^CD8^+^ T cells might be also a heterogenous cell subset with functional diversity. Kim HD et al reported that the tumour-infiltrating CD8^+^ T cells could be subdivided into PD-1-high, PD-1-intermediate, and PD-1-negative subpopulations with distinct gene expression profiles, different exhaustion-related immunophenotypes, and functional capacities [25]. Thus, considering the difference in methods to identify PD-1^+^CD8^+^ T cells, it is possible that the main subsets of PD-1^+^CD8^+^ T cells might be different in Shen et al’s and our study which might account for the inconsistent findings. And furthermore in-depth research about the exact roles for subsets of PD-1^+^CD8^+^ T cells should be conducted in the future.

Several limitations were presented in our current study. First, further studies were required to delve into the biological mechanism of the formation and differentiation of PD-1^+^CD8^+^ T cells in GC. Besides, our usage of the minimal *P* value method to determine the cut-off values may have raised the difficulty of reproducibility. In addition, since our study demonstrated that intratumoral PD-1^+^CD8^+^ T cells is indicative of clinical outcomes, we encouraged future researchers to test whether PD-1^+^CD8^+^ T cells from peripheral blood could serve as a noninvasive biomarker. Also, molecular subtypes might be a confounding factor in survival or other analysis, considering the imbalanced distribution of PD-1^+^CD8^+^ T cells among different molecular subtypes. However, it was not adjusted for the lack of such information based on high-throughput mRNA transcriptome data in ZSHS cohort.

### Reporting summary

Further information on research design is available in the Nature Research Reporting Summary linked to this article.

## Supplementary information

Reporting Summary

Supplementary Figures

## Data Availability

Data and materials generated that are relevant to the results are included in this article. Other data are available from the corresponding author Prof. Xu upon reasonable request.
