# Supplementary material for: Intratumoral PD-1+CD8+ T cells associate poor clinical outcomes and adjuvant chemotherapeutic benefit in gastric cancer
Source: Br J Cancer. 2022 Aug 24;127(9):1709–17. doi: 10.1038/s41416-022-01939-8 (PMC9596411; doi:10.1038/s41416-022-01939-8)
Supplement: Supplementary file 2 — Supplementary Figures [file 41416_2022_1939_MOESM2_ESM.docx]

**ONLINE** **SUPPLEMENTARY** **MATERIAL**

**Contents**

**Supplementary Figure 1. The infiltration of PD1^+^CD8^+^ T cell might optimize the predictive value of prognosis the CD8^+^ T cell.**

**Supplementary Figure 2. Characteristics of PD1^+^CD8^+^ T cell signature expression across molecular subtypes.**

**Supplementary Figure 3. Functional characteristics of GC-infiltrating CD8^+^ T cells in different group of PD1^+^CD8^+^ T cell infiltration.**

**Supplementary Table 1. Antibodies applied for Immunohistochemistry and Flow cytometry.**

**Supplementary Figure** **1. The infiltration of PD1^+^CD8^+^ T cell might optimize the predictive value of prognosis the CD8^+^ T cell.**

**(a)** Kaplan–Meier curve of OS in ZSHS Cohort according to CD8^+^ T cells infiltration. Data were analyzed by log-rank test.

**
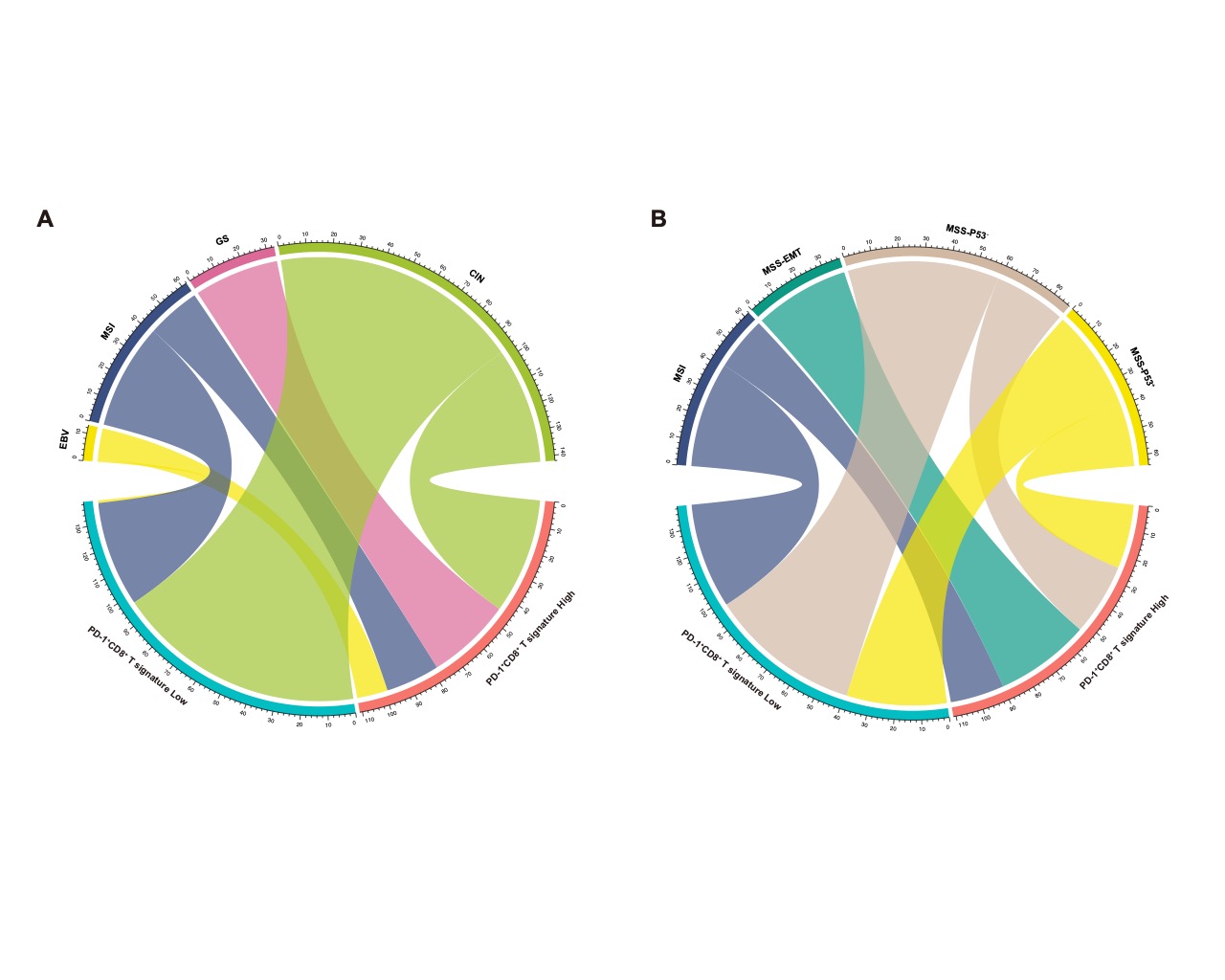
**

**Supplementary Figure 2. Characteristics of PD1^+^CD8^+^ T cell signature expression across molecular subtypes.**

**(a-b)** Chord diagram demonstrated the distribution of different GC molecular subgroups (TCGA classification and ACRG classification) based on PD1^+^CD8^+^ T cell signature level. Patients were from the ACRG cohort (the ACRG cohort, n = 250). CIN, Chromosomal Instability; EBV, EBV-positive; GS, Genomically Stable; MSI, Microsatellite Instable; MSS/EMT, Microsatellite Stable and Epithelial-to-Mesenchymal Transition; MSS/TP53^+^, Microsatellite Stable and Tumor Protein 53 active; MSS/TP53^-^, Microsatellite Stable and Tumor Protein 53 inactive

**
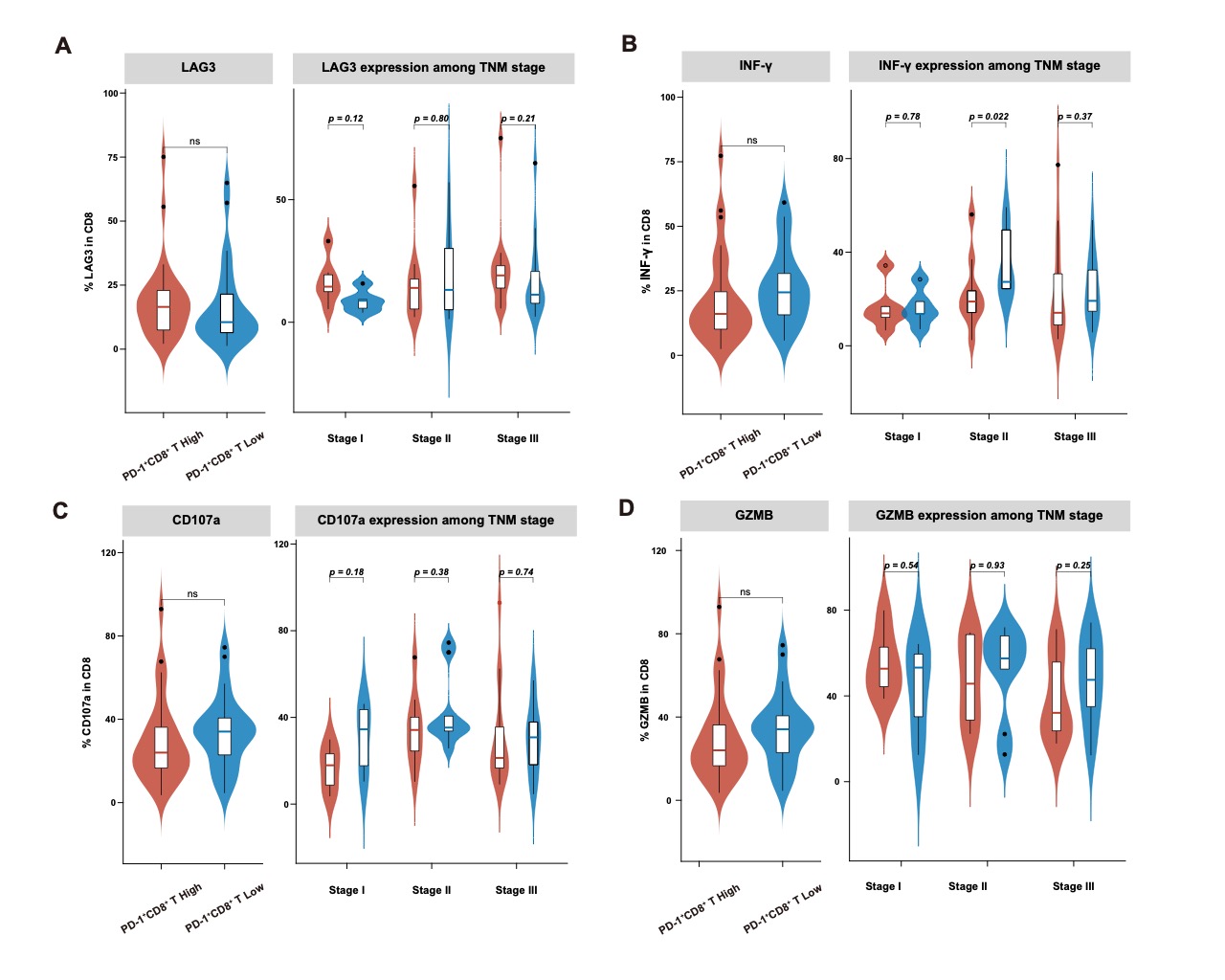
**

**Supplementary Figure 3. Functional characteristics of GC-infiltrating CD8^+^ T cells in different group of PD1^+^CD8^+^ T cell infiltration.**

**(a-d)** Statistical analysis of LAG3^+^, INF-γ^+^, CD107a^+^ and granzyme B^+^ cell percentages in CD8^+^ T lymphocytes among PD-1^+^CD8^+^ T cells high/low abundance subgroups in tumors of GC patients. Data was analyzed by Mann-Whiney U test. Small horizontal lines indicate the Mean (± SD). **P* < 0.05, ***P* < 0.01, ****P* < 0.001, ns refers to not significant. All *P* values presented here were two-tailed. IFN-γ, interferon-γ; GZMB, granzyme B; LAG3, lymphocyte-activation gene 3.

| Supplementary Table 1. Antibodies applied for Immunohistochemistry and Flow cytometry. | | | | | | |
| --- | --- | --- | --- | --- | --- | --- |
| **No.** | **Antibody Name** | **Description** | **Manufacturer** | **Catalog No.** | **Dilution/Dose** | **Application** |
| 1 | Anti-PD1 antibody [EPR4877(2)] | Rabbit monoclonal | Abcam | ab137132 | 1:1000 | IHC |
| 2 | Anti-Human CD8 antibody | Mouse monoclonal | Leica Biosystems | NCL-L-CD8-4B11 | 1:300 | IHC |
| 3 | PE/Cy7 Anti-Human CD45 antibody | Mouse monoclonal | BioLegend | 368532 | 1μl | FCM |
| 4 | FITC Anti-Human CD8 antibody | Mouse monoclonal | BD Biosciences | 555366 | 2μl | FCM |
| 5 | PE Anti-Human CD279 (PD-1) antibody | Mouse monoclonal | BD Biosciences | 560795 | 1μl | FCM |
| 6 | BV 605 Anti-Human CD152 (CTLA-4) antibody | Mouse monoclonal | BioLegend | 369610 | 1μl | FCM |
| 7 | AF 647 Anti-Human TIM-3 (CD366) antibody | Mouse monoclonal | BD Biosciences | 565558 | 1μl | FCM |
| 8 | BV 785 Anti-Human CD223 (LAG-3) antibody | Mouse monoclonal | BioLegend | 369322 | 1μl | FCM |
| 9 | APC-R700 Anti-Human IFN-γ antibody | Mouse monoclonal | BD Biosciences | 564981 | 1μl | FCM |
| 10 | PE Anti-Human GZMB antibody | Mouse monoclonal | eBioscience | MA5-23688 | 1μl | FCM |
| 11 | AF 647 Anti-Human Perforin antibody | Mouse monoclonal | BD Biosciences | 563576 | 1μl | FCM |
| 12 | AF700 Anti-Human 107a antibody | Mouse monoclonal | BD Biosciences | 561340 | 1μl | FCM |
| 13 | Anti-Human CD45 Clone HI30 – APC | Mouse monoclonal | BD Biosciences | 555485 | 1μl | FCM |
| Abbreviation: IHC = Immunohistochemistry; FCM = Flow cytometry. | | | | | | |
